# Challenges Threatening Agricultural Sustainability in Central Asia: Status and Prospect

**DOI:** 10.3390/ijerph19106200

**Published:** 2022-05-19

**Authors:** Yi Qin, Jiawen He, Miao Wei, Xixi Du

**Affiliations:** 1 School of Foreign Languages, China University of Geosciences, Wuhan 430074, China; hjw_1998@cug.edu.cn (J.H.); wm2021@cug.edu.cn (M.W.); duxixi1110@cug.edu.cn (X.D.); 2 School of Public Administration, China University of Geosciences, Wuhan 430074, China; 3 Center for Turkmenistan Studies, China University of Geosciences, Wuhan 430074, China

**Keywords:** sustainable agriculture, comprehensive evaluation, analytic hierarchy process, entropy weight method

## Abstract

Agriculture provides humanity with the most basic products to sustain life and raw materials for production, closely linking human society and nature together. The sustainable development of agriculture, an inevitable choice to maintain long-term social stability, steady economic growth, and ecological security, is the key to the coordinated development of the economy, society, and environment in developing Central Asia economies. We attempted to evaluate the trend of agricultural sustainability in Central Asia between 2002 and 2017 by adopting analytic hierarchy process and entropy weight method in this study. It was found that the overall sustainability level of regional agriculture is rising, which is mainly driven by economic progress, with social and ecological dimensions contributing much less. Accordingly, we advanced four suggestions: enhancing water productivity, optimizing planting techniques, improving agricultural cooperatives, and promoting digital land management to boost the agricultural sustainability of the region.

## 1. Introduction

Derived from the broader paradigm of sustainable development, sustainable agriculture aims to meet the needs of industrial raw materials and food, which are essential for human existence. “Biodynamic farms” and “organic farms” were part of the early idea of sustainable agriculture [1]. A more specific explanation of sustainable agriculture given by the National Food and Agriculture Research Institute (NIFA) is that it aims to protect the environment, expand the natural resource base of the earth, maintain and improve soil fertility.

Based on a multi-faceted goal, sustainable agriculture seeks to increase farm income, promote environmental stewardship, enhance the quality of life for farm families and communities, and increase production to meet people’s needs for food and fiber. Dunlap and others underline that sustainable agriculture should ensure farm income by promoting environmental management and increasing life quality in rural areas [2]. As agriculture connects the ecological environment with economic society, great importance should also be attached to its sustainability and ecological resilience [3].

In a narrow sense, Central Asian countries encompass five member states of the former Soviet Union–Kazakhstan, Kyrgyzstan, Tajikistan, Turkmenistan, and Uzbekistan. The deteriorating ecological environment has hindered the development of agriculture in Central Asia, leading to low crop yields of inferior quality and threatening national food security [4]. As one of the national industries of strategical importance, agriculture is bound up with the natural ecological environment and the economic lifeline and is protected by the respective agricultural policies formulated by the Central Asian states in recent years to keep pace with the global sustainable development trend. A key factor in sustainable development is to ensure that agricultural production is carried out in an environmentally friendly way. For instance, the *Program for the* *Development of the* *Agro-industrial* *Complex in Kazakhstan for 2021–2030* points out that the basic principles of agricultural development in Kazakhstan are balanced and sustainable development, namely, efficient production, environmental protection, and human resources development. The *National* *Development* *Program of Kyrgyz Republic until 2026* highlights the strategic priorities of agricultural development, focusing on environmental conservation and organic production. In 2019, the government of Turkmenistan reorganized relevant departments and established the Ministry of Agriculture and Environmental Protection, aiming at agricultural development, environmental protection, rational utilization of natural resources, analysis of hydrometeorology, food security, and wide application of international practice and modern technology. The fourth chapter of the *Strategy for the Development of Agriculture of the Republic of Uzbekistan for 2020–2030* mainly discusses the rational use of natural resources and environmental protection, expressing the concern about improvement of eco-agricultural practices and modification of standards and mechanisms for the conservation of natural resources. The *National Strategy of the Republic of Tajikistan for the Period up to 2030* stresses one of the principles of agricultural development is to eliminate the impact of human activities on the natural environment and improve the quality of water and soil.

However, despite agricultural sustainability being emphasized in those key documents, most previous studies on the agriculture in Central Asia were stuck in agricultural trade, agricultural planting structure, and consumption of natural resources [5,6,7]. The comprehensive analysis of agriculture from the perspective of economic benefit, ecology, and rural development is inadequate. In this study, the analytic hierarchy process (AHP) and the entropy weight method were used to calculate composite scores of each country and evaluate the sustainability of agriculture in the region. Therefore, the real agricultural situation in Central Asian countries and differences among countries can be revealed thoroughly.

## 2. Materials and Methods

### 2.1. The Study Area

**Figure 1:**
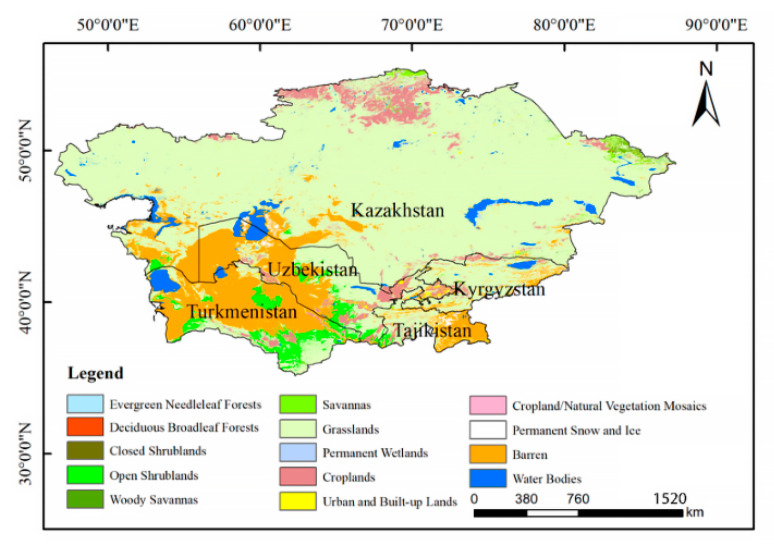
Map of study area. The land cover data was adapted from 500 m Moderate Resolution Imaging Spectroradiometer (MODIS) land cover product (MCD12Q1).

Central Asia, located in the hinterland of the Eurasian continent, mainly includes five Central Asian countries. Its longitude ranges from 45° E to 90° E and its latitude from 35° N to 55° N. This area is relatively high in the southeast, with the Pamirs Plateau in the southeast, the Tianshan Mountains in the east, the Kazakhskiy Melkosopochnik in the north, and the Turan Plain and the Caspian Depression in the middle west (Figure 1). With mountains in the southeast blocking the warm, humid air from the Indian Ocean and the Pacific Ocean, Central Asia enjoys a temperate continental climate with typical features such as little precipitation, dry summers and cold winters. The natural climate and geographical location of Central Asia have severely curbed its agricultural development, especially when the local ecosystem is highly susceptible to environmental perturbations.

Steppe, desert, and semi-desert are the main natural landscapes in this region, largely suitable for the cultivation of drought-resistant and saline-alkali-tolerant plants. Except for the wheat growing areas in northern Kazakhstan, agriculture in this area is dominated by oasis agriculture and is extremely dependent on river irrigation.

### 2.2. Data Source

The data in this paper come from the Food and Agriculture Organization of the United Nations (FAO) database (https://www.fao.org/faostat/en/#data, accessed on 25 February 2022), the United Nations database (UN-data: http://data.un.org, accessed on 2 March 2022), the World bank database (https://data.worldbank.org.cn/, accessed on 23 February 2022), and the FAO-AQUASTAT database (http://www.fao.org/aquastat/en/index.html, accessed on 4 March 2022).

### 2.3. Evaluation Indicator System

In 1999, the Organization for Economic Co-operation and Development (OECD) put forward 39 indicators to measure agricultural sustainability, which mainly include: agricultural financial support, the consumption of water, chemical fertilizers and pesticides, water and soil quality, greenhouse gas emissions, and biodiversity [8]. FAO has also formulated 21 indicators, with collected data mainly from four aspects: ecology, environment, management, and society [9], to monitor the progress of achieving sustainable development goals. In addition, Dantsis, Douma, Giourga, and other scholars divided agricultural sustainability indicators into three main categories: economic, environmental, and social sustainability, showing economic gain, environmental costs, and farmers’ interests and rights, respectively [10]. Therefore, although there are many descriptions about sustainable agriculture, its core lies in ensuring economic sustainability, securing social sustainability (especially rural development), and maintaining environmental sustainability [11]. Based on the three-dimensional evaluation system of economy–society–ecology and availability of data from Central Asia, an evaluation system containing 18 specific indicators for the years from 2002 to 2017 was constructed, as shown in Table 1. In this study, economic efficiency measures the relationship between input of agricultural resources and corresponding economic benefits; social stability describes the long-term viability of the agriculture-producing community; ecological security concerns the interaction between agricultural production activities and natural environment.

### 2.4. Evaluation Methods

#### 2.4.1. Analytic Hierarchy Process (AHP)

The AHP is an analysis method relying on the pairwise comparison of all the indicators. The results reflect the relative importance of the indicators and are based on the subjective judgment of experts in this field [12]. In this study, a total of 10 experts rated the three criterion level indicators according to the Saaty 9-level scale method [13] (Table 2). The 10 experts are researchers in the fields of ecological environment, economy, and agriculture from China’s top universities.

The comparison judgment matrix *A* is established based on the individual preference of experts. $a_{ij}$ represents the relative importance of $a_{i}$ to $a_{j}$, based on the judgment of experts. Both *m* and *n* in matrix *A* are 3, representing 3 indexes in the criterion layer.

$$A={\left[\begin{array}{ccc} a_{11} & \cdots & a_{1j}\\ \vdots & \ddots & \vdots \\ a_{i1} & \cdots & a_{ij} \end{array}\right]}_{m\times n}$$

Weight calculation under single criterion.

(1) The index weight vector obtained by the geometric average method is

$${\bar{H}}_{i}=\frac{{\left(\prod_{j=1}^{n}a_{ij}\right)}^{\frac{1}{n}}}{\sum_{i=1}^{n}{\left(\prod_{j=1}^{n}a_{ij}\right)}^{\frac{1}{n}}}(i=1,2,\cdots ,n);$$

$\bar{H_{i}}$ is the relative weight of index *i* from each one expert.

(2) After calculation, the normalized feature vector is $H_{i}={\left[\bar{H_{1}},\bar{H_{2}},\cdots ,\bar{H_{n}}\right]}^{T}$
(3) Calculate the maximum eigenvalue: $\lambda_{max}=\frac{\sum_{i=1}^{n}{\left(AH\right)}_{i}}{nH_{i}}$
(4) Use the scores of ten experts to construct a new matrix:
  $$\bar{Q}={\left[\begin{array}{ccc} q_{11} & \cdots & q_{1j}\\ \vdots & \ddots & \vdots \\ q_{i1} & \cdots & q_{ij} \end{array}\right]}_{m\times n}(i=1,2,3;j=1,2,\cdots ,10)$$

In matrix $\bar{Q}$, *i* represents the weight of three primary indicators, and *j* represents experts.

(5) Integrate the scores of 10 experts:

$$Q_{i}=\frac{\sum_{j=1}^{n}q_{ij}}{\sum_{\begin{array}{l} 1\leq i\leq m\\ 1\leq j\leq n \end{array}}q_{ij}}(i=1,2,3;j=1,2,\cdots ,10)$$

$Q_{1}$ is the weight of economic efficiency; $Q_{2}$ is the weight of social stability; $Q_{3}$ is the weight of ecological security.

Consistency check.

(1) Calculate the value of consistency index *CI*: $CI=\frac{\lambda_{\max}-n}{n-1}$, the smaller the *CI*, the higher the consistency of the judgment matrix. Consistency reflects the rigorous logic of subjective judgment [12].
(2) Query RI value. The random index RI is introduced to eliminate the difference caused by the order of the matrix. The specific reference values are 0.00, 0.00, 0.58, 0.90, 1.12, and 1.24 for the order from 1 to 6.
(3) Calculate the final consistency ratio (*CR*): $CR=\frac{CI}{RI}$; if the *CR* is less than 0.1, the consistency test will pass. If the *CR* value is 0, it means that there is a perfect level of consistency in the pairwise comparison. If the consistency value is greater than 0.1, then revision must be made in matrix *A* [12].

#### 2.4.2. Entropy Weight Method

The entropy weight method is a way of objectively assigning values according to the discrete degree of the indicators, which is widely used in the field of environmental quality assessment and engineering scheme optimization [14,15]. In information theory, entropy measures the uncertainty or randomness of the index. The greater the variation of the index, the stronger its influence on the whole system and the larger proportion in the comprehensive evaluation [16]. In this study, the entropy weight method was applied to weight each secondary indicator from the index layer between 2002 to 2017, by analyzing the data grouped in periods of five years for each country as a whole. The basic steps are shown below.

Create the raw data matrix. There are *m* samples and *n* indicators in matrix *B*. $x_{ij}$ represents the value of a single country *i* on the indicator *j* in each given year: 2002, 2007, 2012, 2017. In the original data matrix, *m* is 20 and *n* is 18 (Table S3 in the Supplementary Materials).

$$B={\left[\begin{array}{ccc} x_{11} & \cdots & x_{1j}\\ \vdots & \ddots & \vdots \\ x_{i1} & \cdots & x_{ij} \end{array}\right]}_{m\times n}$$

Standardize original matrix. There are different units for different indicators. In order to eliminate the influence of the units, we normalized the data. The indicators involved in this study are mainly positive and negative indicators, which need to be treated differently. The attribute of indicator is shown in Table S2.

The preferred model of higher and better positive indicators: $z_{ij}=\frac{x_{ij}-\min(x_{1j},x_{2j},\cdots ,x_{mj})}{\max(x_{1j},x_{2j},\cdots ,x_{mj})-\min(x_{1j},x_{2j},\cdots ,x_{mj})}(i=1,2,\cdots ,m;j=1,2,\cdots ,n)$.

The preferred model of smaller and better negative indicators: $z_{ij}=\frac{\max(x_{1j},x_{2j},\cdots ,x_{mj})-x_{ij}}{\max(x_{1j},x_{2j},\cdots ,x_{mj})-\min(x_{1j},x_{2j},\cdots ,x_{mj})}(i=1,2,\cdots ,m;j=1,2,\cdots ,n)$.

The normalized matrix is: $Z={\left[\begin{array}{ccc} z_{11} & \cdots & z_{1j}\\ \vdots & \ddots & \vdots \\ z_{i1} & \cdots & z_{\mathrm{ij}} \end{array}\right]}_{m\times n}(0<z_{ij}<1)$.

Calculate information entropy and weight. The information entropy reflects the orderliness of the system. The smaller the information entropy, the larger the dispersion, and the greater the impact of indicator on comprehensive evaluation [16]. Therefore, information entropy is an important tool in entropy calculation process. The formula of information entropy is:

$$e_{j}=-\frac{1}{\ln m}\overset{m}{\underset{i=1}{\sum }}p_{ij}\ln p_{ij}$$

In the formula, $p_{ij}=\frac{z_{ij}}{\sum_{i=1}^{m}z_{ij}}$.

Normalize the information utility value, and then the entropy weight of each index is obtained as follows: $w_{j}=\frac{1-e_{j}}{\sum_{i=1}^{n}\left(1-e_{j}\right)}=\frac{1-e_{j}}{n-\sum_{i=1}^{n}e_{j}}$.

#### 2.4.3. The Combined Weights of Indicator System

The weight of AHP is assigned to the index layer according to the classification standard (economic efficiency, social stability, and ecological security). The operation rules are as follows:

$$\begin{array}{l} f_{j}=\frac{Q_{i}w_{j}}{\sum_{j=1}^{5}w_{j}}(i=1,j=1,2,3,4,5)\\ f_{j}=\frac{Q_{i}w_{j}}{\sum_{j=6}^{10}w_{j}}(i=2,j=6,7,8,9,10)\\ f_{j}=\frac{Q_{i}w_{j}}{\sum_{j=11}^{18}w_{j}}(i=3,j=11,12,13,14,15,16,17,18) \end{array}$$

Among them, $Q_{1}$ is the weight in AHP (weight of indicator in criterion layer), $W_{j}$ is the weight in the entropy weight method (weight of indicator in index layer), and $f_{j}$ is the comprehensive weight of secondary indicator.

Calculate the composite scores of each indicator: $s_{i}=\sum_{j=1}^{n}z_{ij}f_{j}$.

## 3. Results

### 3.1. Weight Calculation Result

The CR values in the evaluation matrix of the ten experts are all less than 0.1, indicating that the data are valid. The weight calculation results of the analytic hierarchy process are shown in Table S1.

According to the scores of the ten experts, the weights of economic efficiency, social stability and ecological security are 0.28, 0.29 and 0.43, respectively.

The weight of each index is calculated according to the information entropy of the actual statistical data. The weights are shown in Table 3.

Combined with AHP and entropy weight method, the comprehensive weight of each index is obtained (Table 3). Please refer to the Supplementary Materials for each indicator data.

### 3.2. Scores Presented by Country

**Figure 2:**
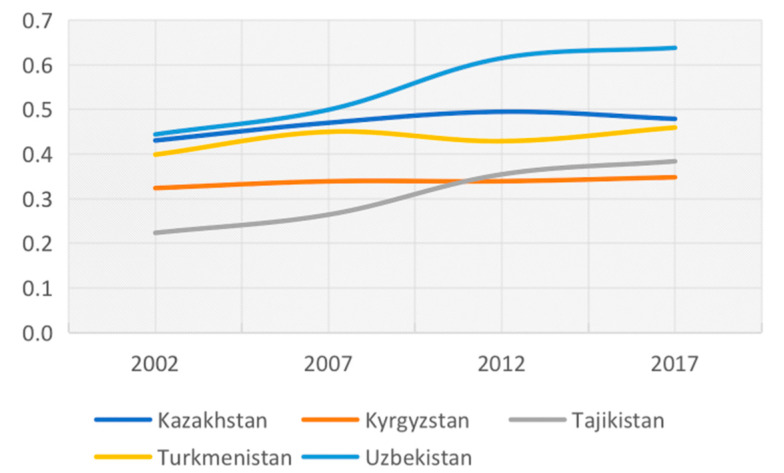
Change of composite scores of agricultural sustainability level in Central Asia.

We ranked the final scores by country and year to get the change in the level of sustainable agricultural development in each country. As shown in Figure 2, the composite scores of sustainable agricultural development in Central Asia from high to low are Uzbekistan, Kazakhstan, Turkmenistan, Tajikistan, and Kyrgyzstan. Uzbekistan showed a marked upward trend, while Kazakhstan, Turkmenistan, and Kyrgyzstan witnessed no significant change over the 15 years from 2002 to 2017.

According to the data in Tables S3 and S5, countries have shown the same trend in some indicators over the 15 years, while there are significant differences in others. From the perspective of economic efficiency, labor productivity in all countries has improved significantly, but agriculture value added share of GDP has been declining. Tajikistan and Uzbekistan surged in land productivity and irrigation water efficiency, while there was almost no fluctuation in the other three countries in land productivity, and Kazakhstan and Kyrgyzstan showed no improvement in irrigation water efficiency. In social stability, the proportion of rural population has dropped slightly, but the per capita arable land has dropped substantially in all countries. Furthermore, the demand for basic drinking water, electricity and sanitation in rural areas was becoming saturated in Central Asia. In terms of ecological security, with the exception of Uzbekistan, water pressure in Central Asian countries has been slightly reduced. Meanwhile, agricultural carbon emissions have decreased, and air quality has improved. Except Kazakhstan, manure consumption increased significantly in other countries, especially in Tajikistan and Uzbekistan. Outside Uzbekistan, the other four countries made little progress in forest area, salinization, drainage, and irrigation rate.

From the comprehensive weight, the top three indicators are per capita arable land, water stress, and land productivity, which just involves the three main factors of agricultural production: labor, land, and water.

## 4. Discussion

### 4.1. Difficulties in Achieving Sustainable Agricultural Development

Stable economic benefits, harmonious agricultural society, and sound ecological environment are the prerequisites for sustainable agricultural development. Over the 15 years from 2002 to 2017, economic efficiency, social stability, and ecological security increased by 54.76%, 19.57%, and 21.02%, respectively (Table 4), which suggests that economic efficiency is the core driver of sustainable agricultural development in Central Asia. The research data shed light on the unshakable position of agriculture in national development—although the agriculture value added share of GDP is shrinking, the relative proportion of agricultural input is still increasing (Table S3). The problems mentioned below continue to slow the pace of agricultural sustainable development in Central Asia.

#### 4.1.1. Inadequate Agricultural Inputs

Agricultural inputs refer to substances used or added in the production process of agricultural products, mainly including consumable inputs such as seeds and chemical fertilizers. Capital inputs are often the more advanced inputs on machinery and technology, such as tractors and irrigation systems. The quality and quantity of agricultural inputs are closely related to the agricultural technology level and industrial production capacity of a country, which are not the strengths of Central Asian countries. First of all, agricultural scientific research investments in Central Asian countries are insufficient, with the total research and experimental expenditure in Central Asia accounting for only 1.4% of GDP in 2018 [17]. Uzbekistan’s agricultural scientific research funds account for only 0.2% of the total agricultural budget [18], and Kazakhstan’s agricultural research investment is less than 1% of GDP [19]. It is unrealistic to achieve the integration of agricultural education, scientific research, and production with the meagre investment in Central Asia. Over the past five years, the supply of native seeds in Kazakhstan has been declining; the import of wheat seeds has increased sevenfold, and that of barley has increased fourfold [19]. More than 80% of the state allocation of the Tajik Academy of Agricultural Sciences is used for the salaries and social security of the researchers. The scientific research laboratory lacks modern equipment, chemical reagents and experimental equipment [20].

**Figure 3:**
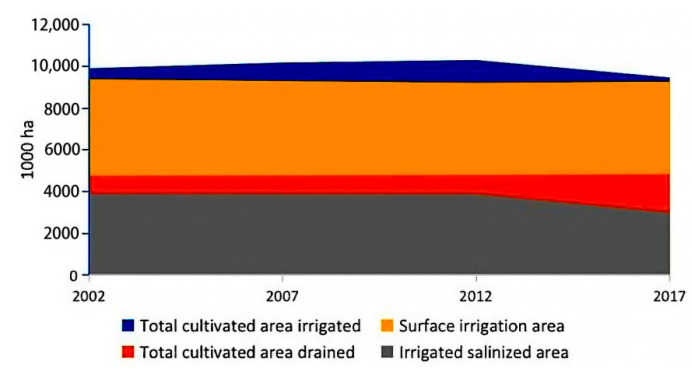
Change of land management in Central Asia.

The irrigation and drainage system is the core of agricultural operations in Central Asia, which, in fact, does not work well [19,21,22,23,24,25,26] and is extremely inadequate, with enormous water losses (up to 60%) in transportation [27]. In addition, the construction of irrigation and drainage systems is progressing slowly (Table S3). Most of the land is surface irrigated, and only half of the irrigated land is equipped with drainage systems (Figure 3). Poor irrigation and drainage systems lead to the downward spiral of land quality in agricultural practices. For instance, irrigation water for agriculture in Turkmenistan is mainly transported through the Karakum Canal, starting from the Amu Darya in the east. However, due to lack of refined management, the Karakum Canal experiences sediment accumulation and clogged drains. Because of the absence of anti-seepage protection and drainage facilities, the flow of salts and minerals between water and farmland soil became unrestricted, causing soil and water pollution. The infiltration of river water and the retention of irrigation water lead to the rise of groundwater level in farmland, which raises salt concentration in the soil surface and aggravates soil salinization [23,27]. In Uzbekistan, the system for collecting and disposing used irrigation water has collapsed, with unhardened surface drainage system playing a leading role. This unsound water supply and drainage system continuously result in a large amount of high-salinity irrigation water leaking into the ground, which accumulates in the shallow middle soil, eventually worsening the physical and chemical properties of soil year by year [24].

#### 4.1.2. Plight of Farmers

Considered as the decisive force for sustainable agricultural development, farmers are a large occupational group in Central Asia. Although there has been a marked improvement in agricultural labor productivity (Table S3), farming is the lowest-paying industry in Central Asia according to the central Asian government’s public income data (Table 5). The *Agrarian Reform Program of the Republic Tajikistan for the Period 2012–2020* once stated that agricultural production in Tajikistan was unprofitable, unproductive, and could not create sustainable jobs, leading to mass migration of the working population [21]. There are untold reasons for farmers’ negativity towards grain production in Central Asia, one of which is strict agricultural management.

Turkmenistan’s agriculture is characterized by state planning and supervision. With the help of the “farmer cooperative”, the government has controlled the planting process of chief crops, such as seed cotton, wheat, barley, sugar beet, and rice. The Turkmenistan government provides farmers with a certain amount of agricultural subsidies, agricultural machinery leasing, and other services through “farmer cooperatives” on the condition that farmers sell their products to the state. Even if international commodity grain prices rise, farmers will not be able to make extra profits. During 2007–2008, national market prices for wheat rose sharply, but the domestic purchase price in Turkmenistan in 2009 was still lower than the previous year at USD 112 per tonne, while Kazakhstan’s wheat price was USD 178 per tonne at the same time [28].

The top-down farming does not take full advantage of farmers’ experience, which is not conductive to increasing production. Under the state-directed system, it is the authority that decides what to grow, when to irrigate, where to sell the crops at what price, with little attention paid to diversified local conditions. Meanwhile, leased land does not give farmers a stable sense of belonging, and therefore, they may overlook soil conservation. In fact, the combination of farmers-initiated bottom-up and government-led top-down approaches better adapts to harsh ecological environment [29], particularly in Central Asia. The ideal state of agricultural production is that the government is responsible for the improvement of unfavorable planting environments, and farmers are committed to the pursuit of high yield and quality products. There is evidence that, with effective organization, risk management, and government oversight, smallholder farmers can become powerful partners in high value specialized production [30].

In Central Asia, small-scale producers often face significant difficulties in accessing agricultural markets and overcoming their size-related disadvantages. In Kazakhstan, individual farmers cannot afford per-unit costs to collect and transport products, and hence, their surplus of high-quality fruits and vegetables can only be sold at lower prices in nearby markets [31]. In Tajikistan, farmers are forced to grow cotton, which would be purchased by cotton monopolies at depressed prices, leaving peasants in the lurch [32]. Some Uzbek farmers now are squeezed by private monopolies, the so-called “cluster” system, which control access to almost everything needed for independent private cotton farming—from agricultural loans, seeds, fertilizers, and fuel to cotton gins and export licenses. Since 2018, an increasing number of Uzbek farmers have not been adequately compensated under contracts with those cluster firms [33]. In Tajikistan, 42% of irrigated land relies on pumping stations due to its location at the upper reaches of the river. About 11% of the land is irrigated in a cascade way, which means irrigation relies heavily on electricity. However, there are tariffs for irrigation water in Tajikistan. During the period from 2000 to 2014 alone, these tariffs increased from 0.6 to 4.32 dirams per cubic meter of water, an increase of 7.2 times (from 0.33 to 0.88 cents in dollars) [32].

Moreover, farmers and rural development in this region have not received necessary and balanced government support. Uzbekistan, for example, has implemented a rural development program that benefits only a few farmers or agricultural producers and completely ignores rural construction [18]. In Kyrgyzstan, safe water supply and basic sanitation service are still not widely accessible in rural areas. Approximately 20% of the rural population does not have access to clean drinking water. Forty percent of rural water is not fully purified, and central sewage treatment capacity only meets the demand for one third of the population [34]. In Kazakhstan, Tajikistan, Uzbekistan, and Kyrgyzstan, the government’s public statistics (data not available for Turkmenistan) rarely use rural areas as a classification criterion. At present, rural construction in Central Asia is still in its initial stage, with only the basic living needs such as water supply and sanitation fully satisfied (Table 6). Accordingly, hardship is driving a massive migration of rural Central Asians to cities at home and abroad in demand of higher incomes and better lives [22,28,32]. Besides, the potentially negative interactions between poverty and ecological degradation has been proved by research [35].

#### 4.1.3. Intensifying Soil and Water Crisis

**Figure 4:**
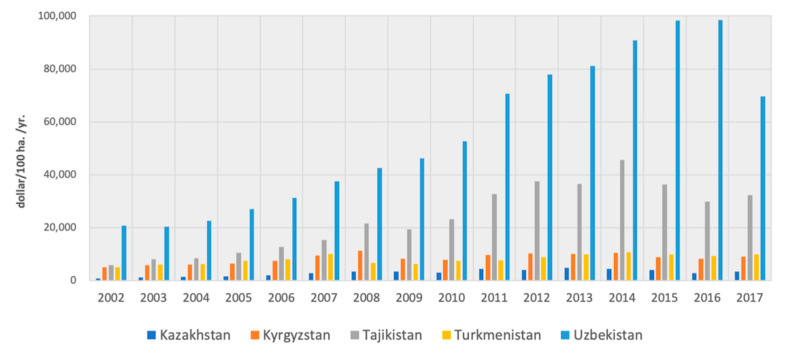
Change of land productivity in Central Asia.

Desert soil, with relatively poor fertility, is the most widely distributed soil type in Central Asia, a temperate desert region [26]. Affected by human activities, the per capita area of cultivated land in Central Asia has been shrinking, and soil fertility has been decreasing. As Figure 4 shows, the land productivity gap among Central Asian countries is huge, and the growth of it in Kazakhstan, Turkmenistan, and Kyrgyzstan is very slow. In Kazakhstan, about 70% of the total area of tillage has low humus content (up to 4%) [36].

**Figure 5:**
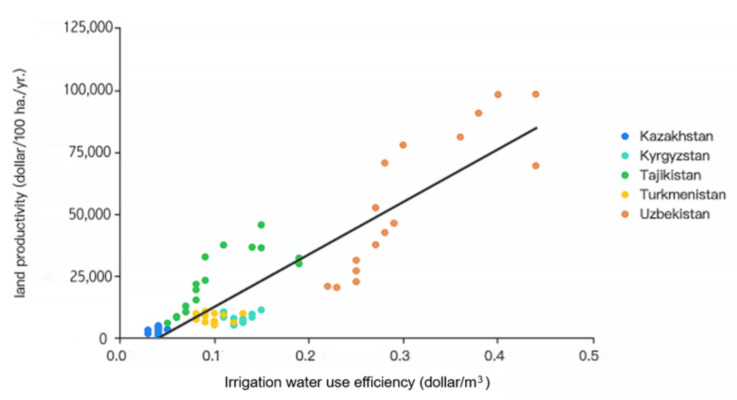
Relationship between irrigation water efficiency and land productivity.

In fact, water and soil efficiency in Central Asia show surprising convergence (Figure 5), making it hard to separate the water and soil issues in Central Asia. Due to the unreasonable use of irrigation water, saline-alkali desertification explains how soil quality declines, and efforts should be intensified to mitigate salinization in Central Asia (Figure 3). Irrigation drainage water, thick with salts, fertilizers, and pesticides, is directly discharged into river, resulting in water pollution. Because of the large-scale withdrawal of water for agricultural irrigation, the water flow from the Amu Darya and Syr Darya into the Aral Sea has plummeted, causing the Aral Sea to shrink. Consequently, drying up of the Aral Sea has led to the formation of large territories of open sea bottom, which fuels the development of powerful sand and salt storms. A conservative estimate of 70 million tons of salt is taken away from the Aral Sea Basin and deposited on 150 to 200 million km^2^ of land annually, severely damaging farms [37]. Water flow is the main driving force for the transportation of salt in Central Asia. Irrigation water not only leaches inorganic salts from deep soil to the surface layer where the plant roots grow but also brings out the salt and chemical substances from the soil with the river water, resulting in secondary pollution and aggravation of the salinization of land. Comparison of the salt inflow and outflow indicates that salt increases from 0.6 to 10 t/ha per year in the middle and lower reaches of the Amu-Darya Basin. The largest part of salt-affected soil and saline water exists in the lower reaches of Amu Darya and Syr Darya Basins, where salinity is one of the main factors threatening food production [38].

### 4.2. Suggestions on Sustainable Development of Agriculture in Central Asia

The ecological core of sustainable agricultural development in Central Asia is rooted in water and soil, and the social core resides in the well-being of farmers. Richard Pomfret also believes that the key to the long-term development of agriculture in Central Asia lies in improving water use efficiency of agricultural irrigation [39]. At present, the efficiency of agricultural resources in Central Asia is extremely low. To achieve the same output, the input of agricultural resources is three to ten times higher than the world average, wasting precious resources [40]. Moreover, agricultural export patterns, dominated by wheat and cotton, have aggravated water stress in the region [5].

Furthermore, rural areas in Central Asia are facing structural problems—relatively high natural population growth, limited employment opportunities and potential food safety crisis [41]. Agricultural sustainable development can not only bring objective economic benefits, but also maintain social stability. In the whole, Central Asian countries can boost sustainable development of agriculture from the following aspects.

#### 4.2.1. Improving Water Productivity

Water erosion is the major reason for soil degradation and salinization in Central Asia, and the anthropogenic activities in Central Asia have exacerbated the deterioration of its ecological environment [42]. In the future, Central Asian countries should continue to actively develop drip irrigation and other water-saving technologies and construct underground irrigation systems, which can accurately transport water and fertilizers to plant roots, significantly reducing evaporation, suppressing weeds, and mitigating the risk of soil salinization. At the same time, reducing losses in the process of water transportation, preventing water pollution, and monitoring water quality changes should be put on the agenda. In the long term, the formulation of a sewage treatment system to reuse purified water for irrigation or recharge rivers can be at the top of the priority list of sustainable development [43].

The improvement of water use efficiency not only relies on the upgrading of equipment but also on reasonable water allocation. The cotton irrigation trial in Turkmenistan has proved that increasing the irrigation water volume from 1 mL/ha to 3 mL/ha can double the cotton yield and improve the quality of cotton. However, increasing the irrigation by three to five times (the national standard irrigation times) does not significantly improve the yield or quality. The potential benefit of reducing irrigation in Turkmenistan by 40% is considerable [44]. Therefore, irrigation water volume and irrigation frequency should be adjusted in line with climatic conditions and the growth stage and water demand of the plant [45,46].

#### 4.2.2. Optimizing Planting Techniques

Adopted as a strategy for production and land use management, conservation agriculture, whose main principles include minimizing mechanical soil disturbance, maintaining constant biomass coverage, and crop diversification, can help countries achieve sustainable agriculture. By shifting to this model, farmers can increase productivity in a sustainable and efficient manner, minimize soil erosion, restore degraded land [47,48], and reduce the use of expensive machinery and fertilizers. In short, conservation agriculture can boost yields while reducing the input of manpower and machinery, which is more efficient than conventional agriculture [48]. The advantages of conservation agriculture have already been proved in Central Asia. It can be seen in the wheat growing areas in northern Kazakhstan that the direct-sown wheat in uncultivated land has higher yields and lower production costs than in cultivated land. Rotation of wheat with other crops can generate additional income and leave plant residue keeping soil moisture and inhibiting weed growth. Conservation agriculture systems containing permanent beds with crop residue retention and diversified crop rotation have turned out to be truly profitable in Central Asia [48,49], since they help to reduce soil salinity [50] and increase land productivity [49] and water efficiency [51].

Phytoremediation agro-technique provides a novel insight into ecological environment restoration. It was found that the cultivation of non-conventional food and bio-energy crops such as pennisetum genus can effectively improve the yield of crops in saline land [52]. Integrated with agroforestry, monoculture crop cultivation contributes to diversifying land use and improving soil fertility [53]. Unfortunately, although these new planting technologies with higher social, economic, and ecological benefits have won wide recognition from agronomists, they have not been applied on a large scale in Central Asia.

#### 4.2.3. Improving Agricultural Cooperatives

Cooperatives seek to ‘optimize’ outcomes for a range of stakeholders so that people can have access to goods and services without being exploited. Therefore, economic, social, and environmental sustainability should be overarching motivations and justifications for the growing cooperative movement [54]. Small farms with higher production efficiency, which play a dominate role in Central Asia, have to rely on agricultural cooperatives for production, but the farmers in post-socialist economies (including Central Asia) have little incentive to participate in cooperatives [55,56].

While Kazakhstan and Kyrgyzstan privatize agricultural land and allow the free flow, merger, and concentration of land, agricultural land has not been fully privatized in the other three countries, where land ownership still belongs to the state, and farmers can only lease it through agricultural cooperatives [57]. Apart from managing the circulation of land, cooperatives are vehicle for obtaining agricultural materials as well. A notable feature of agricultural cooperatives in Central Asia is its top-down, state-controlled form. Empirical evidence shows that this model, with less empowerment for farmers [55], is not as successful as expected [56,58].

Improved agricultural cooperatives, with the combination of both top-down and bottom-up approaches in the region, are hence needed to help small-scale producers increase productivity, benefit from economies of scale, enjoy greater bargaining power with other actors in the supply chain, and gain better access to extension services and technology transfer systems [8]. They may also inform farmers of market dynamics both at home and abroad and offer them technical and financial assistance to protect their income [59].

#### 4.2.4. Promoting Digital Land Management

Widespread land degradation, soil salinization, and extensive farming could render agricultural productivity ineffective. The integrated use of geographical information systems (GIS), satellite imaging, and computational modeling techniques is an efficient means of monitoring changes of land cover and distribution of crops. In addition, land productivity can be measured and calculated by using satellite archive imagery. In Central Asia, better knowledge of dynamics and spatial patterns of cropland use intensity and productivity can be used as baseline for optimizing planting structure [60] and water distribution [61]. Satellite remote sensing can also monitor the emergence of abandoned agricultural land and assess its productivity [62], allowing owners of nearby plots to quickly restore the land and avoid land wastage. Predictably, agriculture in Central Asia is developing towards intelligent agriculture, characterized by accurate inputs to crops and soils.

## 5. Conclusions

AHP and entropy weight method to quantify the process of agricultural sustainable development were used in this study in Central Asia over the 15 years from 2002 to 2017. Overall, agricultural sustainability in Central Asia has increased, especially the economic sustainability, leaving much room for improvement in society and ecology domains. To compensate for the limitation attributable to the unavailability of certain data from Central Asian countries, we draw on a wider range of materials to provide detailed potential risks of primary agricultural production in Central Asia.

The weight results show that the traditional water and soil problems are inveterate problems in the process of achieving sustainable agricultural development in Central Asia, and the newly emerged man–land contradiction also plays a vital role in this regard. It was found that capital investment is insufficient for ecological restoration, and the agro-ecological development of Central Asian countries has not yet broken through the limitations of the environmental background. Furthermore, the opportunities for an upward and superior quality of life remain rather detached from the rural population. Advanced technology and targeted management policies, therefore, may be the best choice for promoting ecological restoration, agricultural productivity, and farmers living and working conditions in Central Asia.

## Figures and Tables

**Table 1:** Evaluation indicator of agricultural sustainability.

Criterion Layer	Index Layer	Explanation	Unit	Attribute	Data Source
Economic efficiency	Labor productivity	Per capita gross output value of agriculture, forestry, animal husbandry, and fishery.	dollar/capita/yr.	Positive	FAO database
	Land productivity	Per unit yield gross output value of agriculture, forestry, animal husbandry, and fishery.	dollar/100 ha./yr.	Positive	FAO database
	Agriculture value added share of GDP	The annual added value of agriculture in the proportion of GDP.	%	Positive	FAO database
	Agricultural input	The ratio of gross fixed capital formation of agriculture to GDP.	%	Negative	FAO database
	Irrigation water use efficiency	The ratio of the net income of crops to the water used to generate those benefits.	dollar/m^3^	Positive	FAO-AQUASTAT database
Social stability	The proportion of rural population	The proportion of rural population in the total population.	%	Negative	World bank database
	Per capita arable land	The ratio of cultivated land area to total population.	ha./capita	Positive	FAO database
	Electrification rate	The proportion of rural population with access to electricity.	%	Positive	UN-data
	Drinking water safety	The proportion of rural population with access to safe drinking-water.	%	Positive	UN-data
	Sanitary conditions	The proportion of rural people using at least basic sanitation services.	%	Positive	UN-data
Ecological security	Carbon intensity of agricultural production	Total amount of agricultural GHG emissions per unit of agricultural added value.	kg CO_2_eq/dollar	Negative	FAO database
	Water stress	The ratio of annual freshwater withdrawals of agriculture to total freshwater withdrawal.	%	Negative	FAO-AQUASTAT database
	Manure consumption	The amount of manure consumed per unit farmland.	kg/ha.	Positive	FAO database
	Forest coverage	The ratio of forest area to total land area.	%	Positive	FAO database
	Salinization	The ratio of salinized land area to irrigated farmland area.	%	Negative	FAO-AQUASTAT database
	Irrigation rate	The proportion of harvested irrigated crop area.	%	Positive	FAO-AQUASTAT database
	Drainage rate	The proportion of cultivated area drained.	%	Positive	FAO-AQUASTAT database
	PM2.5 air pollution	Mean annual exposure of PM2.5 air pollution.	micrograms per cubic meter	Negative	UN-data

**Table 2:** Saaty 9-level scale score sheet.

Intensity of Importance	Definition	Explanation
1	Equal importance	Two activities contribute equally to the objective
3	Moderate importance	Experience and judgment slightly favor one activity over another
5	Strong importance	Experience and judgment strongly favor one activity over another
7	Very strong or demonstrated importance	An activity is favored very strongly over another; its dominance demonstrated in practice
9	Extreme importance	The evidence favoring one activity over another is of the highest possible order of affirmation
Reciprocals of above	If activity i has one of the above nonzero numbers assigned to it when compared with activity j, then j has the reciprocal value when compared with i.	A reasonable assumption
Rationals	Ratios arising from the scale	If consistency were to be forced by obtaining *n* numerical values to span the matrix.

**Table 3:** Comprehensive weight results.

Criterion Layer	AHP Weight	Index Layer	Entropy Weight	Comprehensive Weight
Economic efficiency	0.28	Labor productivity	0.05	0.04
		Land productivity	0.10	0.09
		Agriculture value added share of GDP	0.06	0.05
		Agricultural input	0.02	0.02
		Irrigation water use efficiency	0.09	0.08
Social stability	0.29	The proportion of rural population	0.05	0.06
		Per capita arable land	0.14	0.16
		Electrification rate	0.02	0.02
		Drinking water safety	0.02	0.02
		Sanitary conditions	0.03	0.03
Ecological security	0.43	Carbon intensity of agricultural production	0.02	0.02
		Water stress	0.10	0.10
		Manure consumption	0.05	0.05
		Forest coverage	0.06	0.06
		Salinization	0.04	0.04
		Irrigation rate	0.04	0.04
		Drainage rate	0.07	0.07
		PM_2.5_ air pollution	0.03	0.03

**Table 4:** Comprehensive scores change of three indicators of criterion layer in Central Asia.

Criterion Layer	Year				Growth Rate
	2002	2007	2012	2017	
Economic efficiency	0.335	0.409	0.499	0.519	54.76%
Social stability	0.551	0.578	0.629	0.659	19.57%
Ecological security	0.933	1.034	1.104	1.129	21.02%

**Table 5:** Comparison between monthly per capita agricultural income and national income in Central Asia in 2020. (Unit: dollar).

Country	Agricultural Income	National Income
Kazakhstan	296.67	485.27
Kyrgyzstan	127.80	230.98
Tajikistan	46.63	112.85

**Table 6:** Representative information on rural areas in Central Asia.

Year	Electrification Rate (%)	Drinking Water Safety (%)	Sanitary Conditions (%)
2002	99.55	74.80	93.17
2007	99.41	77.89	95.28
2012	99.65	81.28	97.50
2017	99.87	83.58	98.83

## Supplementary Materials

The following supporting information can be downloaded at: https://www.mdpi.com/article/10.3390/ijerph19106200/s1, Table S1: Weighting results of AHP; Table S2: Entropy weighting results; Table S3: Initial data; Table S4: Normalized data; Table S5: Detail scores.
